# Pre-Task Prefrontal Activation during Cognitive Processes in Aging: A Near-Infrared Spectroscopy Study

**DOI:** 10.1371/journal.pone.0098779

**Published:** 2014-06-04

**Authors:** Yumi Oboshi, Mitsuru Kikuchi, Yoshiyuki Shimizu, Yuko Yoshimura, Hirotoshi Hiraishi, Hiroyuki Okada, Yasuhiro Magata, Yasuomi Ouchi

**Affiliations:** 1 Department of Biofunctional Imaging, Medical Photonics Research Center, Hamamatsu University School of Medicine, Hamamatsu, Japan; 2 Department of Occupational Therapy, Faculty of Health and Medical Sciences, Tokoha University, Hamamatsu, Japan; 3 Research Center for Child Mental Development, Kanazawa University, Kanazawa, Japan; 4 Central Research Laboratory, Hamamatsu Photonics KK, Hamamatsu, Japan; 5 Department of Molecular Imaging, Medical Photonics Research Center, Hamamatsu University School of Medicine, Hamamatsu, Japan; University of Rome, Italy

## Abstract

**Purpose:**

Cognitive processing generally deteriorates as people age. Recent neuroimaging studies have shown that the prefrontal cortex (PFC) is involved in human-specific behavior, such as preparing for future actions and prospective memory; hence, the PFC may be voluntarily activated even during the “resting” condition that precedes task execution. The purpose of the present study was to investigate changes in voluntary pre-task activation as a result of aging using a paradigm that includes a longer intertrial interval (e.g., 30 sec) than has been used in previous studies.

**Methods:**

A total of 120 cognitively normal adults (young: 60, old: 60) participated in this near-infrared spectroscopy (NIRS) study. All subjects performed 6 repetitions of the working memory task, which included a 30 sec resting period and a 28.8 sec task period. The resting period was divided into baseline and pre-task (preT) periods, and the task period was divided into early easy task (eT) and late difficult task (dT) periods. We then normalized the data, analyzed the magnitude of task-related NIRS responses in each period and compared the results between groups using an analysis of variance test.

**Results:**

Statistical analyses revealed a significant interaction between group × optode location × period, in which hemodynamic responses in the PFC during the preT period were smaller in the elderly in than young adults. By contrast, during the task period, the hemodynamic responses were higher in the lateral PFC in the elderly than in young adults. Spearman's rank correlation analysis showed a positive correlation between hemodynamic changes during the preT period in the PFC and correct answer ratios in both groups.

**Conclusions:**

These findings suggest that more pre-task activation in the anterior PFC is related to better cognitive performance in humans. Thus, a reduction in this activation might partly explain cognitive decline in the elderly.

## Introduction

The anterior prefrontal cortex (PFC) is involved in human-specific behavior, such as preparing for future information processing [1]–[3], preparing for future actions [4], thinking about the future [5], and prospective memory [6]–[8]. It has also been reported that the frontopolar cortex is recruited to encode the outcome of a decision before the answer is noticed [9]. Thus, the anterior PFC plays an important role in preparing for future events. The region that is considered the anterior PFC in this study includes Brodmann Area 10, which is sometimes referred to as the frontopolar or rostral PFC.

Several studies have used functional magnetic resonance imaging (fMRI) [10], [11] and electroencephalography (EEG) [12]–[14] to examine the effects of aging on prefrontal activity during the pre-task anticipation period. However, inconsistent results due to different methodological strategies (e.g., recording devices such as fMRI and EEG, or applications of simple repeated and switching tasks) have made the effect of aging on prefrontal activity dynamics unclear. Some of these previous results have suggested an age-related neural under-recruitment in proactive cognitive control and an age-related neural over-recruitment in reactive cognitive control [10], [11], [14]. Exploiting the strength of EEG (i.e., high time resolution), Berchicci et al. demonstrated that older subjects prepared for the action with greater anticipation and higher cost, as indicated by an earlier latency onset and amplitude of movement, related to cortical potentials in the PFC [12], [13]. These previous studies chiefly examined brain activity using a relatively short intertrial interval (ITI) (i.e., 2.5–7.5 sec) in which time-based prospective memory load might be small. In addition, some of these studies did not employ a fixed ITI because they focused on different aspects (e.g., task switching load) in which brain activity induced by time-based prospective memory load might be a confounding factor. Thus, the aging effect on prefrontal recruitment during time-based prospective memory load with a fixed ITI is poorly understood, and the effect of prefrontal activity dynamics on cognitive performance also remains unexplored.

The purpose of the current study was, therefore, to uncover the mechanism of age-related altered prefrontal recruitment during anticipation by measuring brain hemodynamics during working memory tasks performed repeatedly with long and fixed ITIs (i.e., 30 sec) in young and elderly adults. We hypothesized that elderly adults would have less prefrontal activity during the anticipation period than young adults and that the less prefrontal preparatory activity during the anticipation period would correlate with lower cognitive performance in elderly subjects. Despite an inability to measure hemodynamic changes in the whole brain, NIRS allows us to measure hemodynamic changes in a calm, natural environment that is similar to that of daily life. This natural environment made for conditions free of psychological stress and anxiety, both of which affect brain activities in the PFC [15], [16] and performance in working memory tasks [17], [18].

## Materials and Methods

### Ethics statement

The present study was approved by the Ethics Committee of Hamamatsu Medical Center, and all procedures were performed in accordance with the Declaration of Helsinki. Written informed consent was obtained from all participants prior to enrollment.

### Participants

Sixty cognitively normal younger subjects (mean age: 21.7±3.3 years old, 33 women and 27 men) and 60 cognitively normal elderly subjects (mean age: 71.0±6.4 years old, 31 women and 29 men) took part in this study (Table 1). Fifty-eight young and 56 elderly subjects used chopsticks to eat daily and have written with the right hand since early childhood. The remaining 2 young and 4 old subjects were ambidextrous and used the left hand in sports, such as tennis and baseball, but usually used chopsticks and wrote with the right hand. The duration of school education of the elderly group was significantly shorter than that of the young group (Mann-Whitney U test, u = 194.5, *P*<0.005) (Table 1). This difference was mainly due to limited access to higher education for elderly people in conjunction with their surrounding sociological environment. However, as shown by the clinical tests that follow, the education difference itself is unlikely to have affected their performance levels. Several psychological tests (Mini-Mental State Examination (MMSE), Frontal Assessment Battery test (FAB), Word generation test, Cubic copy test) were performed to verify that all participants were cognitively normal. No subjects had serious vision problems, a history of dementia, brain injury, or psychiatric disorders.

**Table 1:** Participant characteristics.

	Younger	Elderly
Age (years)	21.7±3.3 (range: 19–33)	71.0±6.4 (59–87)
Sex (female/male)	33/27	31/29
Education (years)	14.5±1.5 (range: 13–19)	10.7±2.3 (6–18)**
FAB score	17.5±0.8	16.4±1.3**
MMSE score	N/A	28.6±1.8
Word generation	14.8±4.5	14.6±4.2 N.S.
Cubic copy	No errors	No errors

Data are expressed as mean ± standard deviation.

**P<0.005. N.S., no significant differences; FAB, Frontal Assessment Battery (18 points represents a perfect score); MMSE, Mini-mental state examination; N/A, not applicable (Not all younger subjects were evaluated with MMSE). Word generation: the average number of words generated in one minute.

### NIRS measurement

**Figure 1:**
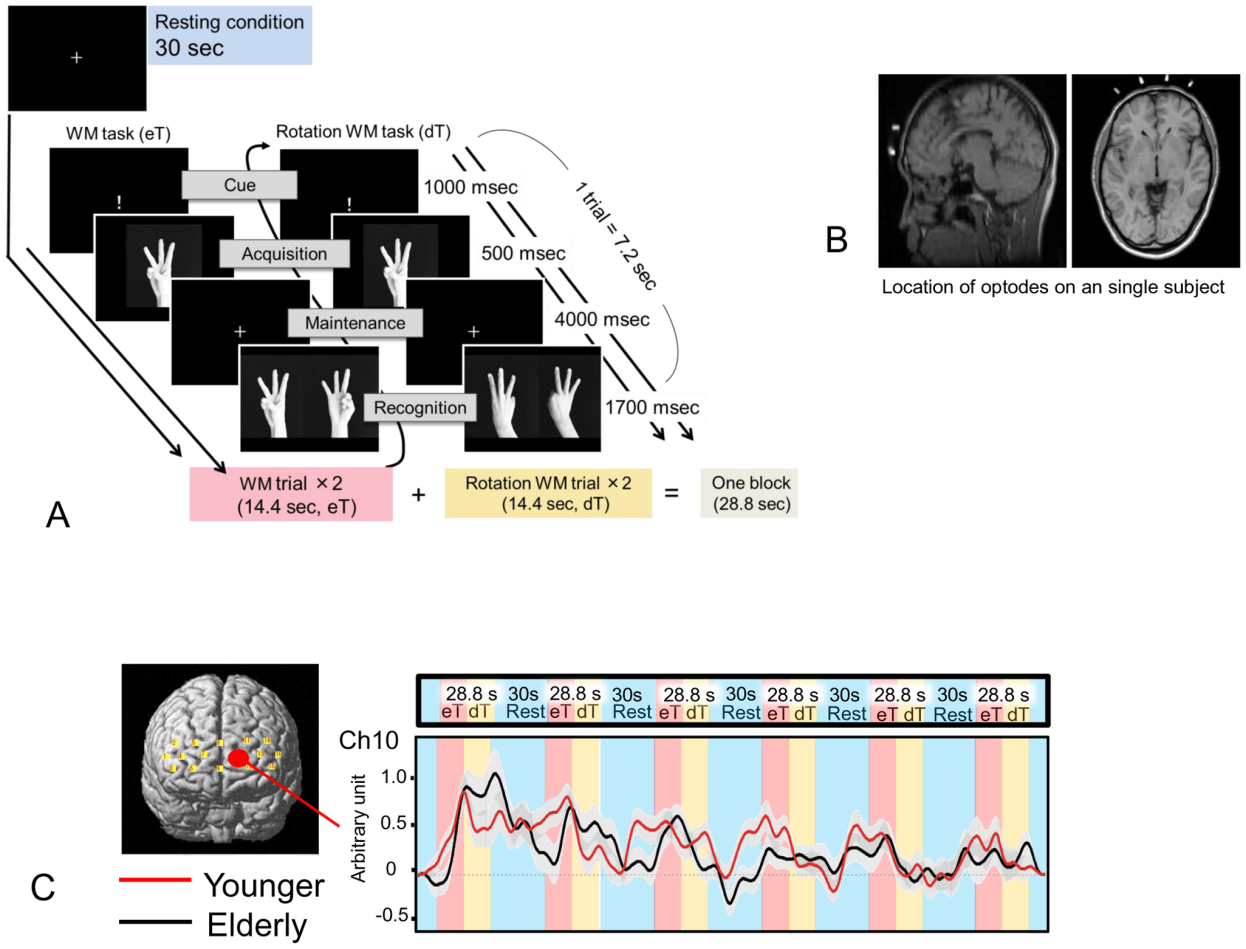
Task and hemodynamic responses. (**A**) A sequence of rest and two types of visual working memory (WM) tasks: non-flipped object (hand) images for easy tasks (eTs) and flipped images for difficult tasks (dTs). (**B**) Locations of optodes of the OEG-16 device on an MRI. (**C**) An example of hemodynamic responses (grand average waveforms of Δ[oxy-Hb] changes) during a session in which 6 rest and 6 task (eT and dT) blocks were included for younger and elderly adults. The gray band represents ± one standard error.

We used a 16-channel NIRS device utilizing two types of infrared light, 770 nm and 840 nm (OEG-16, Spectratech Inc.) [19]. The probe consisted of two rows of photodiodes (3 emitters and 3 detectors for each row). The distance between emitter and detector was 3 cm, allowing for hemodynamic measurements 2–3 cm from the skin [20]. The sampling rate for the recording was 1.526 Hz. Hemoglobin changes depended on the light path length, and changes in the concentrations of oxygenated hemoglobin (oxy-Hb), deoxygenated hemoglobin (deoxy-Hb), and total hemoglobin (total-Hb) were calculated [21]. The midpoint of a photodiode pair (an emitter and a detector) in the center of the bottom row was located at Fpz according to the international 10/20 system for electroencephalography. We examined the positions of the emitter and detector probes of the current NIRS system in some subjects using MRI (Hitachi Medical Co., MRP-7000AD, 0.3T) and confirmed that the photodiodes in the center of the bottom row were located on the rostrum of the PFC (Fig. 1B, 1C).

### Working memory task

We used our previous visual working memory task [22] modified for NIRS measurements. Visual tasks were presented on a liquid crystal screen in front of subjects during NIRS recordings. As shown in Fig. 1C, one experimental session consisted of 6 task blocks with regular inter-task resting intervals of 30-sec periods. The task block (28.8 sec) was divided into two periods (each 14.4 sec). Two types of working memory task were used; as shown in Fig. 1A, the easy visual working memory task (eT) was completed during the first period (14.4 sec), and the difficult visual working memory with mental rotation task (dT) was completed during the second period (14.4 sec). In the first period (eT), in the recognition phase, subjects were asked to select the picture with the same hand (non-flipped images) as was presented in the acquisition phase. During the latter period (dT), in the recognition phase, subjects were asked to select the picture with the same hand flipped. In the acquisition phase, the palm was facing the subject, and in the recognition phase, the hand was flipped so that the rear was visible. Both periods (14.4 sec) consisted of two working memory trials. As shown in Fig. 1A, each trial lasted for 7.2 sec and consisted of a start cue “!” (presented for 1000 milliseconds), an acquisition phase (500 milliseconds) to memorize the hand shape, a maintenance phase (4000 milliseconds) with “a white cross” to keep the image in mind and a recognition phase (1700 milliseconds) to select the correct picture from two pictures as quickly as possible by pressing the corresponding button attached to the controller device the subject was holding with both hands.

Before beginning a session, researchers provided the participants with instructions on how to perform the task, and each subject was fully informed of the sequence of tasks and the length of the inter-task resting periods (i.e., 30 sec). During inter-task resting periods, subjects were instructed not to move their head and to stare attentively at a blue cross on the monitor without thinking.

### Data analysis and statistics

The mean reaction time (RT) in the working memory task was analyzed using an unpaired t-test between the younger and elderly groups, and a non-parametric Mann-Whitney U test was used to compare psychological test scores and values of the correct answer ratio (scores of each subject/full scores) between groups. Statistical significance was assumed to be *P*<0.05. All these statistics were performed with SPSS ver.19 for Windows.

**Figure 2:**
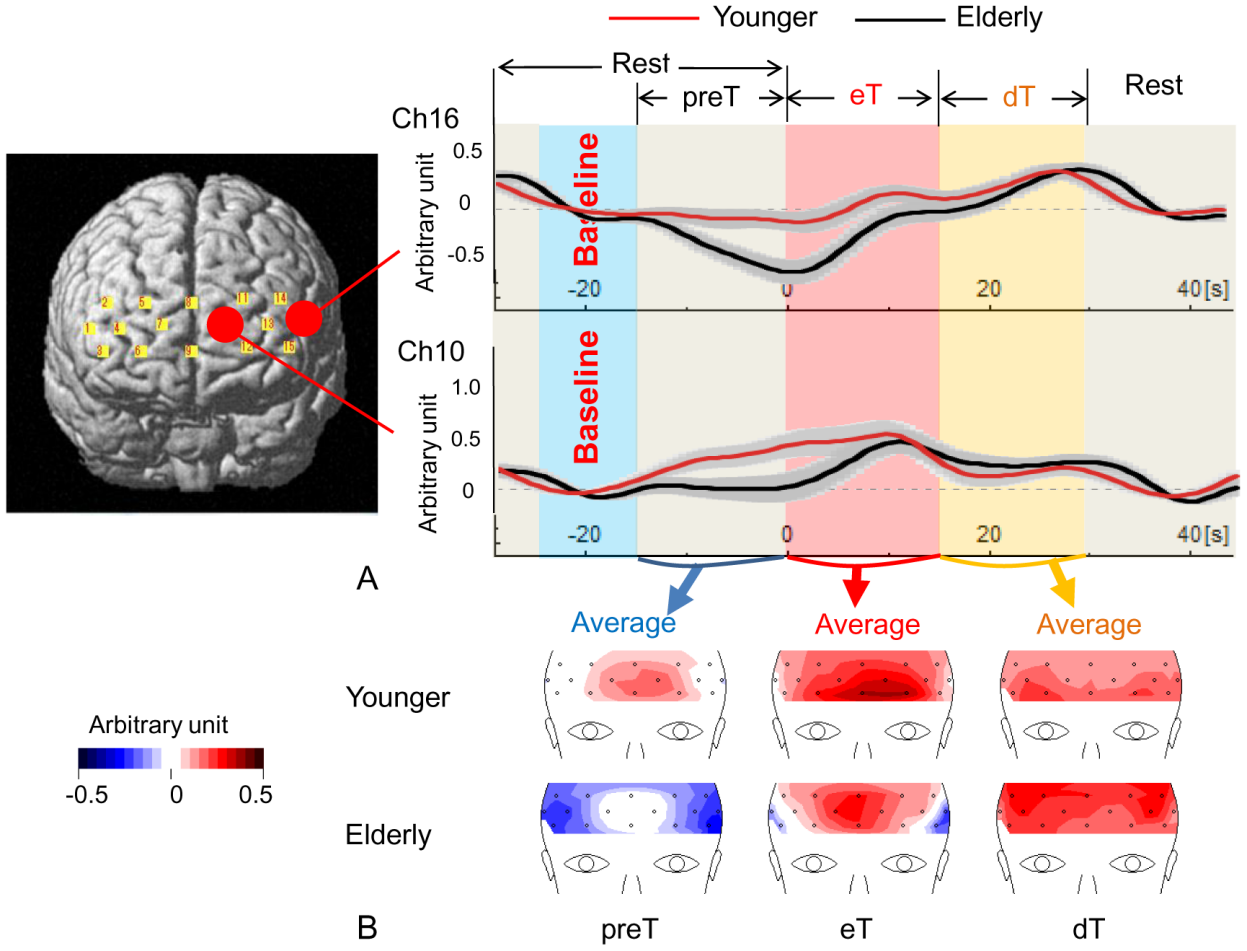
Design scheme and topography of mean hemodynamic responses. (**A**) An example of hemodynamic responses occurring in the medial and lateral optodes in both groups. The baseline activity was set during 25 sec to 15 sec before the task start during the resting period. The 6 experimental blocks were averaged after the baseline correction. The upper scheme shows grand average waveforms in one channel corresponding to the left lateral PFC. The lower scheme indicates grand average waveforms in one channel corresponding to the anterior PFC. In those schemes, red and black lines denote grand average waveforms from 60 younger subjects and 60 elderly subjects, respectively. The gray band represents ± one standard error. (**B**) Grand mean topographic images of time-averaged values of Δ[oxy-Hb] during the preT, eT and dT periods by a Brain Vision Analyzer. Upper and lower pictures are results from younger and elderly subjects, respectively. Note that the Δ[oxy-Hb] in the lateral PFC tended to increase after the onset of the WM task in both groups, whereas, in the anterior PFC, the Δ[oxy-Hb] level had already elevated during the pre-task period in younger subjects.

We used a Brain Vision Analyzer (Brain Products GmbH, Gilching, Germany) and Matlab software (MathWorks, Natick MA) to analyze the NIRS data. To examine brain hemodynamic changes in the PFC region covered by the NIRS device, we evaluated Δ[oxy-Hb] changes quantitatively throughout the whole experimental session on 16 channels. To minimize physiological brain spontaneous oscillation and correct drift artifacts, a low cut filter of 0.1 Hz and global direct current trend correction [23] were applied for the whole 384-sec NIRS data set (i.e., the whole experimental session). The values of each sampling point were first normalized by dividing each sampling point by its standard deviation calculated from the whole experimental period (i.e., 384 sec) for each channel. Then, the normalized data during the whole period were averaged for each group (i.e., grand average data). Because our main interest was to examine age-related pre-task changes of Δ[oxy-Hb], we chose the level of Δ[oxy-Hb] during the resting period 25 to 15 seconds prior to the task period as the baseline Δ[oxy-Hb] level. Consistent with the fact that different subregions of the PFC subserve different cognitive processes [24]–[31], a variety of responses in each channel was found within this area. As shown in Fig. 2A, for example, different patterns were observed in the medial and lateral PFC regions, showing that the levels of Δ[oxy-Hb] seemed to be higher in the medial region during the preT and eT periods in the younger subjects, while these levels were elevated in the lateral PFC during the later dT phase in the elderly subjects. Mean Δ[oxy-Hb] values in each period were analyzed by a three-way ANOVA [group (young, old), period (preT, eT, dT) and location (channel 1–16)] using SPSS. Because significant interactions were found among groups, periods, and channels, a post-hoc two-way ANOVA [group and period] was performed for each channel. To examine the difference of preT Δ[oxy-Hb] values between the younger and elderly subjects, an additional analysis using an unpaired t-test [group] was performed for preT Δ[oxy-Hb] values in each channel.

Furthermore, we calculated the hemodynamic changes between dT and eT (i.e., dT - eT) for each subject and for each channel and performed a two-way ANOVA [group and location]. Because significant interactions were found between groups and channels, a post-hoc unpaired t-test [group] was performed for Δ[oxy-Hb] values (dT - eT) in each channel. Statistical significance was defined as *P*<0.05.

### Correlation analysis

We calculated the Pearson's correlation coefficient between mean Δ[oxy-Hb] values in preT periods (i.e., preT - baseline periods) and the hemodynamic changes (i.e., dT - eT) during the working memory task. Statistical significance was defined as *P*<0.05.

Because the assumption of normality for the correct answer ratio is violated in a Shapiro-Wilk test, Spearman's rank correlation coefficient was calculated for correlation analyses between physiological and psychological data. Specifically, it was calculated for mean Δ[oxy-Hb] values (preT, eT and dT periods) and psychological performance data within each group and for the hemodynamic changes during working memory task (i.e., dT - eT) and psychological performance data within each group. Statistical significance was defined as *P*<0.05.

## Results

### Cognitive performance

As shown in Table 2, the unpaired t-test revealed that the mean RT was significantly longer in elderly subjects than in younger subjects (t = 11.126, *P*<0.005). The Mann-Whitney U test revealed that the levels of the correct answer ratio (u = 935.500, *P*<0.005) (Table 2) and FAB score (Table 1) (u = 859.000, *P*<0.005) were significantly lower in the elderly subjects than in the younger subjects.

**Table 2:** Results of cognitive performance.

	Younger	Elderly
Reaction time (ms)	852.91±128.11	1147.86±160.47**
Correct answer ratio	0.95±0.48	0.89±0.92**

Data are expressed as mean ± standard deviation.

**P<0.005.

Correct answer ratio: answer score divided by the full score (24 points).

### Brain responses during the pre-task and task periods

**Figure 3:**
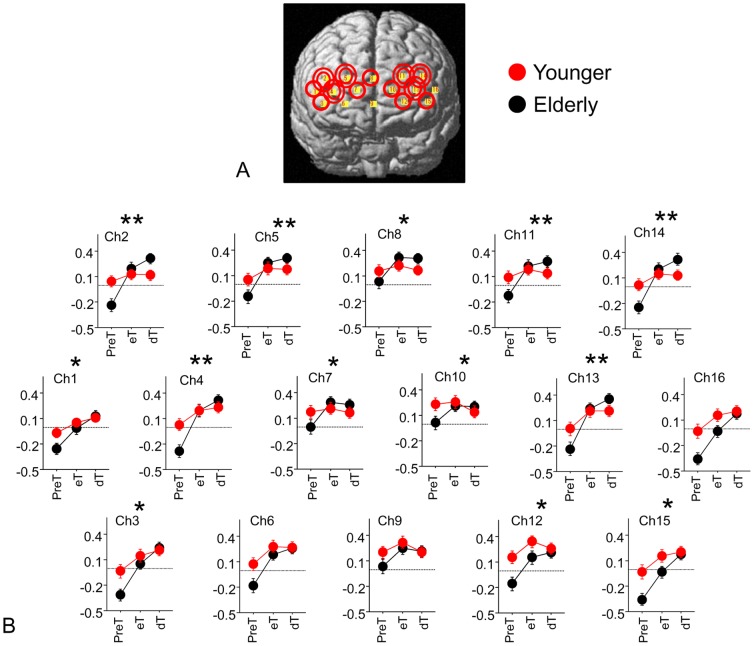
Statistical comparisons of hemodynamic changes between younger and elderly subjects. (**A**) Significant interactions (group × period) are observed in the areas with red circles; single circle: *P*<0.05; and double circles: *P*<0.005. (**B**) Time-averaged levels of Δ[oxy-Hb] in all channels. Because our main interest was to examine age-related pre-task changes of Δ[oxy-Hb] relative to the baseline condition, we chose the level of Δ[oxy-Hb] during the resting period 25 to 15 seconds prior to the task period as the baseline Δ[oxy-Hb] level (horizontal dash line). The ordinate in each diagram indicates the level of normalized Δ[oxy-Hb]. *P<0.05, **P<0.005.

The three-way ANOVA for mean Δ[oxy-Hb] values (difference from baseline period) revealed significant main effects for two factors: i.e., “Location” (F (15, 1770) = 11.07, *P*<0.005), and “Period” (F (2, 236) = 24.59, *P*<0.005). In addition, significant interaction effects were observed for “Group × Location” (F (15, 1770) = 2.81, *P*<0.005), “Group × Period” (F (2, 236) = 6.86, *P*<0.005), “Location × Period” (F (30, 3540) = 11.38, *P*<0.005) and “Group × Location × Period” (F (30, 3540) = 2.15, *P*<0.005). As shown in Table 3 and Fig. 3, the post-hoc two-way ANOVA for each channel showed the significant main effect of “Period” in the following channels: 1, 2, 3, 4, 5, 6, 7, 8, 9, 11, 12, 13, 14, 15 and 16 (F (2, 236) = 5.62–39.54, *P*<0.005). There was an effect of “Group” in the following channels: 12, 15 and 16 (F (1, 118) = 4.37–4.54, *P*<0.05). A significant “Group” and “Period” interaction was found in the following channels: 1, 3, 7, 8, 10, 12 and 15 (F (2, 236) = 3.34–5.02, *P*<0.05) and in 2, 4, 5, 11, 13 and 14 (F (2, 236) = 6.41–14.69, *P*<0.005).

**Table 3:** Results of the hemodynamic responses by two-way ANOVA in all channels.

	Group	Period	G×P		Group	Period	G×P
Ch 1	1.24	25.21**	3.34*	Ch 9	0.77	5.62**	1.58
Ch 2	0.01	26.09**	14.69**	Ch 10	0.60	2.58	4.03*
Ch 3	2.12	31.49**	4.44*	Ch 11	0.03	12.53**	6.41**
Ch 4	0.91	39.54**	9.48**	Ch 12	4.37*	15.48**	3.40*
Ch 5	0.00	21.58**	6.55**	Ch 13	0.07	36.67**	7.45**
Ch 6	2.29	22.36**	2.71	Ch 14	0.00	27.52**	11.19**
Ch 7	0.00	5.89**	4.59*	Ch 15	4.42*	34.21**	5.02*
Ch 8	0.20	6.85**	3.90*	Ch 16	4.54*	35.24**	2.16

F values were presented. G×P, interaction between group and period.

*P<0.05,

**P<0.005.

As shown in Table 4, the unpaired t-test between two groups for each channel showed significantly higher Δ[oxy-Hb] levels during the preT period in younger subjects in the following channels: 1, 3, 6, 10, 11, 12, 13, 14 and 16 (t = −2.77 to −2.01, *P*<0.05) and in 2, 4, 15 (t = −3.02 to −2.88, *P*<0.005) and a tendency of higher Δ[oxy-Hb] level in channels 7, 8, 9. This indicated that the cerebral blood flow during the preT period was augmented more in the PFC in the younger than in elderly adults.

**Table 4:** Comparison of the Δ[oxy-Hb] levels during the pre-task period between younger and elderly subject using unpaired t-test in all channels.

	Younger vs. Elderly		Younger vs. Elderly
Ch 1	−2.06*	Ch 9	−1.53
Ch 2	−2.88**	Ch 10	−2.02*
Ch 3	−2.61*	Ch 11	−2.01*
Ch 4	−3.02**	Ch 12	−2.77*
Ch 5	−1.83	Ch 13	−2.17*
Ch 6	−2.23*	Ch 14	−2.52*
Ch 7	−1.57	Ch 15	−2.98**
Ch 8	−1.03	Ch 16	−2.52*

T-values were presented. Negative values means higher value in younger compared to elderly subject.

*P<0.05,

**P<0.005.

The two-way ANOVA for the hemodynamic changes between dT and eT (i.e., dT - eT) revealed significant main effects for one factor; i.e., “Location” (F (15, 1770) = 32.06, *P*<0.005). In addition, a significant interaction effect was observed for “Group × Location” (F (15, 1770) = 1.90, *P*<0.05). As shown in Table 5, the post-hoc unpaired t-test between the groups for each channel showed significantly higher values in elderly subjects in the following channels: 2, 3, 12, 14, 15 and 16 (t = 2.06–2.80, *P*<0.05).

**Table 5:** Comparison of the hemodynamic change (dT - eT) during the working memory task between younger and elderly subject using unpaired t-test in all channels.

	Younger vs. Elderly		Younger vs. Elderly
Ch 1	1.59	Ch 9	1.32
Ch 2	2.14*	Ch 10	1.77
Ch 3	2.06*	Ch 11	1.66
Ch 4	1.66	Ch 12	2.36*
Ch 5	1.07	Ch 13	1.97
Ch 6	1.48	Ch 14	2.16*
Ch 7	0.52	Ch 15	2.80*
Ch 8	0.73	Ch 16	2.18*

T-values were presented. Positive values means higher value in elderly compared to younger subject.

*P<0.05

In summary, these results indicated statistically significant increases in the Δ[oxy-Hb] levels during the preT in the younger group in the channels over the PFC and during the dT in the elderly group in the channels covering the lateral part of the PFC.

### Correlation analyses on changes in brain responses between the pre-task and task execution periods

As shown in Table 6, in both the younger and elderly subjects, there were strong negative correlations between mean Δ[oxy-Hb] values in preT periods (i.e., preT - baseline periods) and the hemodynamic changes (i.e., dT - eT) during the working memory task in all channels.

**Table 6:** Results of correlation coefficient between Δ[oxy-Hb] levels during the pre-task period and the hemodynamic change (dT - eT) during the working memory task.

	Younger	Elderly		Younger	Elderly
Ch 1	−0.663**	−0.880**	Ch 9	−0.830**	−0.821**
Ch 2	−0.756**	−0.821**	Ch 10	−0.844**	−0.824**
Ch 3	−0.841**	−0.826**	Ch 11	−0.838**	−0.795**
Ch 4	−0.773**	−0.809**	Ch 12	−0.868**	−0.834**
Ch 5	−0.831**	−0.824**	Ch 13	−0.800**	−0.808**
Ch 6	−0.827**	−0.842**	Ch 14	−0.786**	−0.793**
Ch 7	−0.810**	−0.831**	Ch 15	−0.806**	−0.756**
Ch 8	−0.838**	−0.814**	Ch 16	−0.759**	−0.785**

Correlation coefficient (r) values were presented.

**P<0.005.

### Correlation analyses between brain responses and performance data

**Figure 4:**
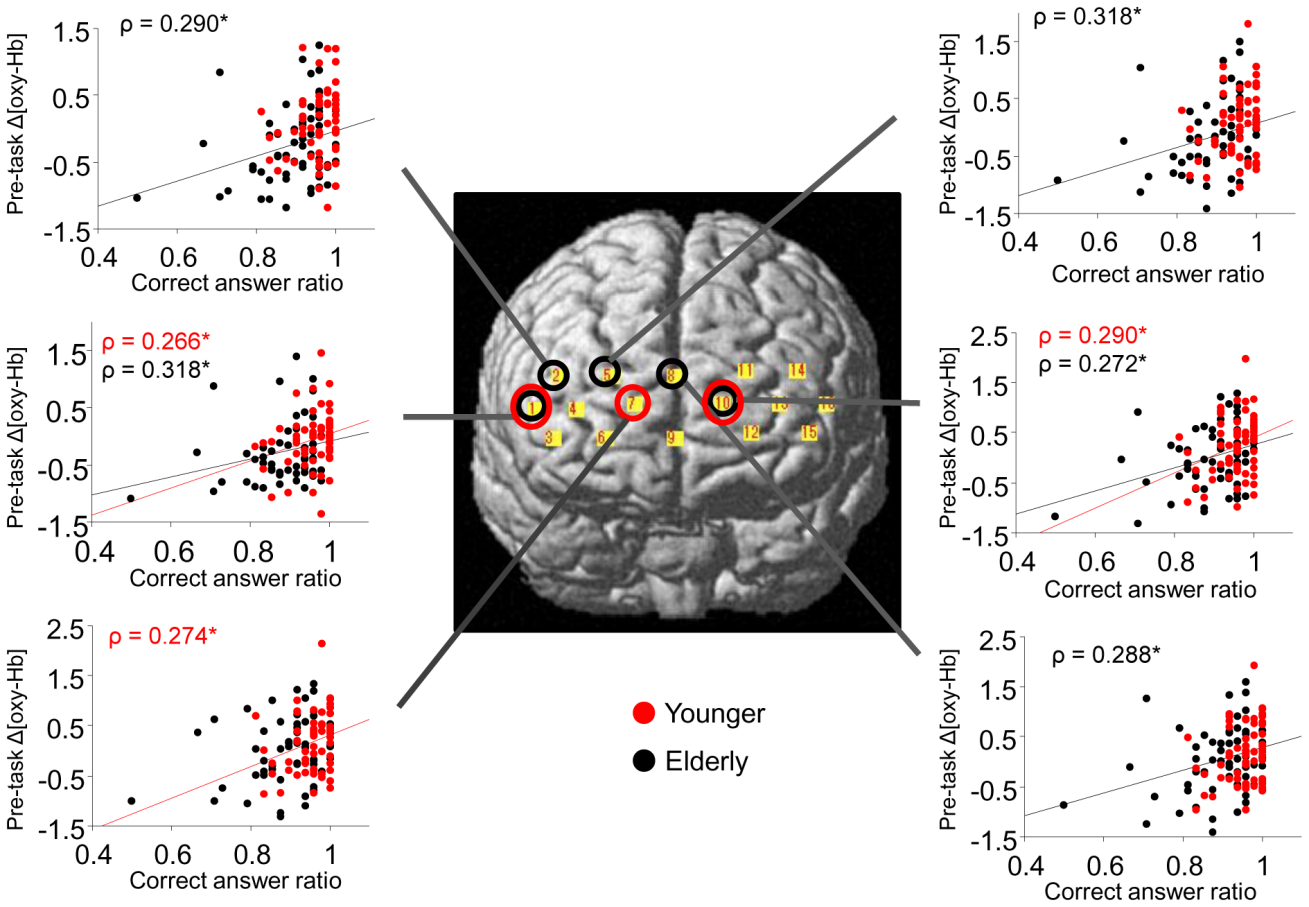
Correlations between cognitive performance and hemodynamic responses. Open red and black circles indicate the channels where significant correlations between pre-task Δ[oxy-Hb] values and correct answer ratios were present in younger and elderly subjects, respectively. In the scattergrams, significant correlations were found in younger (red line) and elderly (black line) subjects. The ordinate indicates a normalized pre-task Δ[oxy-Hb] value. *P<0.05.

As shown in Table 7 and Fig. 4, Spearman's rank correlation coefficient revealed a significant positive correlation between the mean Δ[oxy-Hb] change during the pre-task period and the correct answer ratio in channels 1, 7 and 10 (*P*<0.05) in the younger subjects and in channels 1, 2, 5, 8 and 10 (*P*<0.05) in the elderly subjects. A weak, non-significant correlation was observed for FAB scores in some channels (data not shown). In addition, no significant correlation was found for the two task periods (eT and dT). The present correlation results indicate that better working memory performance is associated with greater pre-task brain responses in the medial part of the PFC in the younger group and in the medial to right-lateral part of the PFC in the elderly group.

**Table 7:** Results of correlation coefficient between Δ[oxy-Hb] levels during the pre-task period and correct answer ratio in all channels.

	Younger	Elderly		Younger	Elderly
Ch 1	0.266*	0.318*	Ch 9	0.102	0.186
Ch 2	0.204	0.290*	Ch 10	0.290*	0.272*
Ch 3	0.124	0.147	Ch 11	0.214	0.250
Ch 4	0.210	0.175	Ch 12	0.046	0.217
Ch 5	0.193	0.318*	Ch 13	0.135	0.200
Ch 6	0.156	0.141	Ch 14	0.177	0.187
Ch 7	0.274*	0.164	Ch 15	0.101	0.228
Ch 8	0.224	0.288*	Ch 16	0.135	0.217

Correlation coefficient (ρ) values were presented.

*P<0.05.

As shown in Table 8, an increase in brain blood flow during the latter half of the working memory tasks (i.e., dT - eT) was associated with poor working memory performance in the elderly subjects in the following channels: 1, 2, 3, 4, 5, 6, 8, 10 and 11 (*P*<0.05), most of which were different channels from the ones in which significantly higher values were observed in the elderly subjects compared with the younger subjects (i.e., channels: 2, 3, 12, 14, 15, 16) (Table 5). In the younger subjects, there were no significant correlations between the cerebral blood flow changes during the working memory task (i.e., dT - eT) and working memory performance in any channel.

**Table 8:** Results of correlation coefficient between the hemodynamic change (dT - eT) during the working memory task and correct answer ratio in all channels.

	Younger	Elderly		Younger	Elderly
Ch 1	−0.118	−0.292*	Ch 9	0.017	−0.206
Ch 2	−0.151	−0.313*	Ch 10	−0.125	−0.267*
Ch 3	−0.007	−0.265*	Ch 11	−0.183	−0.328*
Ch 4	−0.057	−0.334*	Ch 12	0.072	−0.147
Ch 5	−0.130	−0.339*	Ch 13	−0.101	−0.197
Ch 6	−0.032	−0.264*	Ch 14	−0.158	−0.248
Ch 7	−0.146	−0.229	Ch 15	0.023	−0.156
Ch 8	−0.113	−0.325*	Ch 16	0.073	−0.204

Correlation coefficient (ρ) values were presented.

*P<0.05.

## Discussion

Using a NIRS device, we found significantly increased brain responses during the resting period immediately before cognitive execution of visual working memory tasks in the PFC of younger adults. Although this pre-task augmentation in the anterior PFC was not present in the elderly adults, a gradual increase in the response was found in the lateral PFC during the task period, as shown in Fig. 3. Comparison with the elderly group, the younger group showed significantly higher Δ[oxy-Hb] levels in the PFC during the preT period. These results demonstrated an age-related difference in brain responses during the pre-task resting period in a sequence of cognitive process.

### Pre-task brain activation in the PFC

In the present study, pre-task brain activation in the PFC was highlighted as a possible neurophysiological biomarker for aging. Interviews following the NIRS study suggested that the participants remained alert while a blue cross was presented on the screen before task initiation by engaging either in prospective thinking about the subsequent stimuli. Previous fMRI studies showed that cognitive processes for prospective memory or task setting are accompanied by anterior prefrontal activation [1], [8], [32]. Prospective memory is considered to be the ability to perform an intended action after a delay period filled with unrelated activity [6], and the task setting is assumed to be the configuration of cognitive processes maintained actively for subsequent task performance [3]. Many pieces of evidence confirm that the anterior PFC is an important mediator of those cognitive processes [1], [3], [7], [8], [32]–[35]. Stroke patients with lesions in the anterior PFC made mistakes in time-based prospective memory trials but did not make errors in basic attention, detection or inhibition [36]. In the present study, the pre-task brain activation was found in the anterior PFC region only in the younger group. As shown in Fig. 1C and Fig. 2A, in this region there seemed to be periodic augmentation of brain responses corresponding to the condition-shift periods (i.e., from preT to eT, from eT to dT), suggesting that the anterior PFC might also subserve unconscious preparatory activation. This possibility was supported by the results from previous fMRI studies with a task-switching task [37] and a branching task [38], in which the medial, not the lateral, part of the PFC was activated while the participants predicted the timing of a task switch and the onset of a subsequent task.

### Aging effect on hemodynamics and cognitive performance

In both younger and elderly subjects, higher values in oxy-Hb change during the working memory task (i.e., dT - eT) were strongly associated with lower mean Δ[oxy-Hb] values in the preT periods (Table 6). This result suggests that such characteristic hemodynamics are not a specific phenomenon related to aging. In contrast, the finding of lower cerebral blood flow during the preT period in most PFC areas combined with higher cerebral blood flow during the latter half of a working memory task (i.e., positive values for dT - eT) in some lateral prefrontal areas may reflect an aging effect (Table 4, 5).

Additionally, in elderly subjects, a higher cognitive performance was associated with a higher mean Δ[oxy-Hb] level during the preT period (Table 7) at the channels corresponding to the anterior PFC and right lateral PFC; higher performance was also associated with a lower mean Δ[oxy-Hb] level during the latter half of working memory task (i.e., negative values for dT - eT) (Table 8). These channels did not necessarily overlap with the regions where the cerebral blood flow was significantly different between the elderly and younger subjects (Table 4, 5). In other words, age alone cannot predict the brain area implicated in cognitive decline. It might be possible that the anterior PFC is activated to compensate for cognitive decline due to aging. A further study with a longer ITI protocol is necessary to examine whether these findings in the anterior PFC of the elderly were primarily related to the brain preparatory processes during pre-task periods or to the general delay of the brain hemodynamic response.

### Limitations

First, because NIRS covers only a small area, the measured hemodynamic responses were limited in the PFC areas, and only events on the cortical surfaces, as opposed to those in the deep medial and orbitofrontal regions, could be measured. However, our interest in the present study was to observe hemodynamic fluctuations during a pre-task period in the anterior PFC, which is considered a neurophysiologically important region for task-reaction cognitive processes. Second, we adopted a sequential paradigm in which the order of a relatively easy task (eT period) and a difficult task (dT period) was not counterbalanced as shown in Fig. 1A. However, because the order of task contents (shapes of hand) during each period was random, neither a recollection effect nor a practice effect was likely in the present study. Third, not all participants underwent an MRI scan before the NIRS measurement; therefore, the exact brain region focused on by the NIRS device was unknown. Although there may be a location error in this NIRS study, the sufficiently large number of subjects (60 adults in each group) allowed data analysis to blur the location error, similar to the image normalization performed in fMRI and PET studies. Fourth, there is a possibility that processing effort, arousal or attentional levels could explain the increase in the oxy-Hb level during eT and dT in both groups because such factors might affect the blood flow after the task periods. An additional study using tasks of various difficulties may be necessary to rule out this possibility. As such, in the present study, we cannot distinguish working memory-induced brain activities from prospective memory-induced pre-activation occurring in the PFC.

## Conclusions

In conclusion, we showed for the first time that pre-task brain responses in the PFC during cognitive task execution are significantly smaller in elderly adults than in young adults, suggesting that one of the neurophysiological features of aging may be reduced activity in this brain area when preparing for upcoming cognitive demand (preparatory capacity). A greater degree of PFC pre-task activity may yield better cognitive performance in humans regardless of age.
